# The complete mitochondrial genome of *Silurus grahami* Regan, 1907 (Siluriformes: Siluridae), a native catfish in Fuxian Lake

**DOI:** 10.1080/23802359.2021.1884024

**Published:** 2021-03-11

**Authors:** Junjie Wu, Chunyun Lei, Jingxia Zhao, Fangpeng Jin, Haitao Gao, Shiwei Fu, Rui Zhou, Yongxin Luo, Yun Leng, Shaowei Xue, Wenkui Zhang, Guanghua Li

**Affiliations:** Yunnan Institute of Fishery Sciences Research, Kunming, China

**Keywords:** *Silurus grahami*, mitochondrial genome, phylogenetic analysis

## Abstract

In this study, the whole mitochondrial genome of *Silurus grahami* was reported to be 16,518 bp in length, including 13 protein-coding genes, two ribosomal RNAs, 22 transfer RNAs, and one control region. The phylogenetic analysis based on 13 protein-coding genes showed that *S. grahami* was sister to clade of *S. meridionalis* and *S. lanzhouensis*. A total of 81 bases differences were identified in COI barcoding region, which could be used for species identification in catfish.

*Silurus grahami* belongs to Siluriformes, Siluridae, *Silurus*. It is a benthic catfish and only distributes in Fuxian Lake area in Yunnan province of China (Chen 2013). Although there was several published research on the mitochondrial genome of *S. glanis* (Vittas et al. 2011), *S. lanzhouensis* (Wang et al. 2012), *S. asotus* (Zeng et al. 2011), and other *Silurus* fish, there were no available literature about *S. grahami*. It is necessary to make more research about native catfish of Yunnan. In this study, we completed the complete mitochondrial genome sequencing and performed the phylogenetic analysis of the genus *Silurus*.

The biological specimen of *S. grahami* was sampled on the east coast of Fuxian Lake (4°34′21.43″N, 102°56′53.88″E) and stored in Specimen Bank of Yunnan Institute of Fishery Sciences Research (Junjie Wu, wujunjie2007@yeah.net) under the voucher number: 20200428001. The DNA extraction was based on the ammonium acetate precipitation method and followed previous study (Rivero et al. 2006). The PCR primers were designed according to the complete mitochondrial genome of *S. lanzhouensis* (GenBank accession no. JF895472.1) and *S. meridionalis* (GenBank accession no. HQ907992.1). Finally, the PCR products were performed by Sanger two directional sequencing. The products of Sanger sequencing were assembled by using SeqMan Lasergene Version 7.1 (Swindell and Plasterer 1997). The complete mitochondrial DNA sequence of *S. grahami* was 16,518 bp in length and consisted of 13 protein-coding genes, two ribosomal RNAs (rRNAs), 22 transfer RNAs (tRNAs), and one D-loop control region. The base composition of mitochondrial genome of *S. grahami* was A (30.1%), T (25.1%), C (28.5%), and G (16.2%), respectively. The A + T and G + C content was 55.1% and 44.9%, respectively.

Most of the genes are the heavy-strand protein-coding genes, ND6 and eight tRNAs (tRNA-Gln, tRNA-Ala, tRNA-Asn, tRNA-Cys, tRNA-Tyr, tRNA-Ser, tRNA-Glu, and tRNA-Pro) were in light-strand. Comparing with closely related species, the identity of nucleotides was 94.39% to *S. meridionalis* (HQ907992.1), 93.95% to *S. lithophilus* (LC520058.1), 93.88% to *S. asotus* (AP012022.1), and 93.25% to *S. lanzhouensis* (JF895472.1). A total of 81 bases substitution (no insertion or deletion) were identified in the COI gene, which was widely used as a ‘Fish barcoding’ region for species identification in bony fish.

To identify the phylogenetic relationship of *Silurus grahami* in the family of *Silurus*, 14 published mitochondrial genome sequences of *Silurus* were downloaded and the 13 protein-coding genes were analyzed in jModeltest 2.1.3 for substitution model evaluation (Posada 2008), the RAxML GUI 2.0 was used for rebuilding the Maximum Likelihood tree (Silvestro and Michalak 2012). The ML tree showed that *Silurus grahami* was phylogenetically closest to the clade of *Silurus meridionalis* and *Silurus lanzhouensis* (Figure 1).

**Figure 1.**
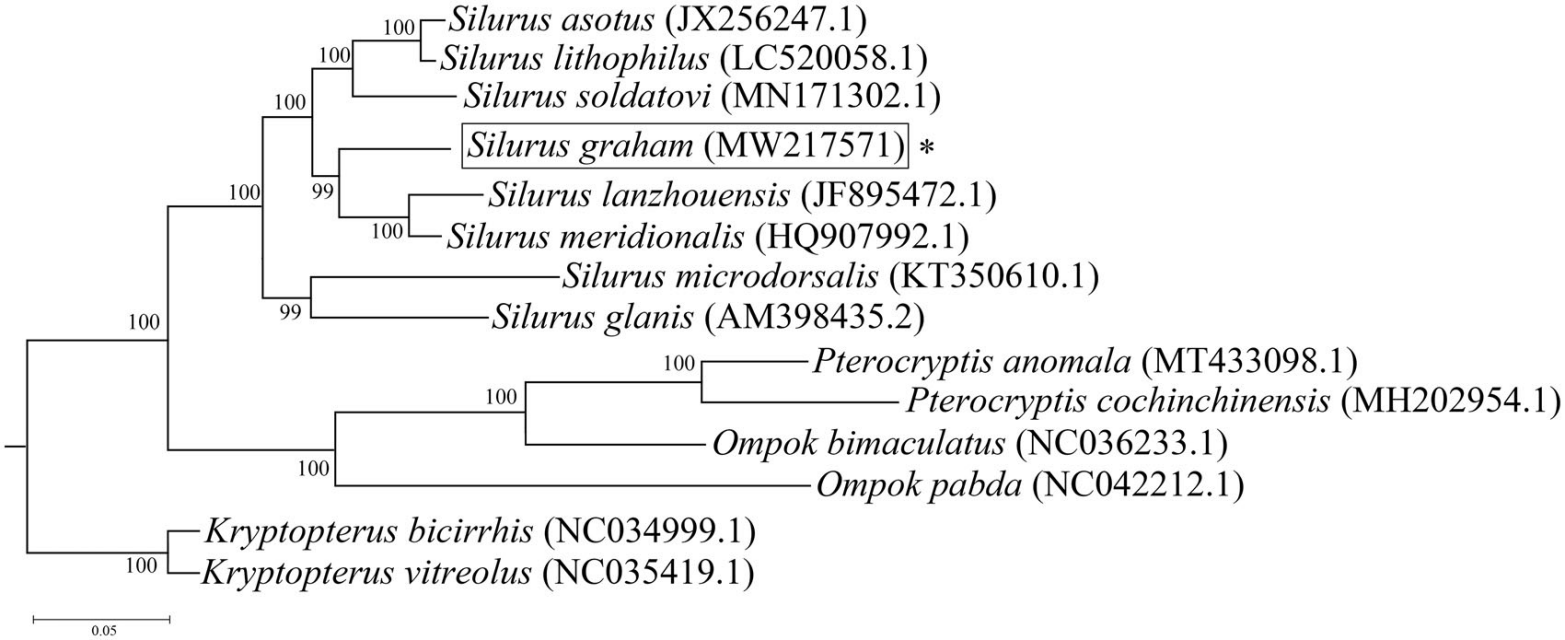
The maximum-likelihood (ML) tree in species of Siluridae based on 14 mitochondrial protein-coding genes. The ‘*’ represented *Silurus grahami* in our study.

## Data Availability

The genome sequence data that support the findings of this study are openly available in GenBank of NCBI at https://www.ncbi.nlm.nih.gov under the accession no. MW217571.
